# Pace-and-ablate in a flash: Ablation of the atrioventricular node with pulsed field ablation

**DOI:** 10.1016/j.hrcr.2025.06.012

**Published:** 2025-06-16

**Authors:** Ven Gee Lim, Faizel Osman

**Affiliations:** Department of Cardiology, University Hospitals Coventry and Warwickshire (UHCW) NHS Trust, Coventry, United Kingdom, Institute for Cardio-Metabolic Medicine (ICMM), UHCW, Coventry, United Kingdom, and Warwick Medical School, University of Warwick, Coventry, United Kingdom

**Keywords:** Pulsed field ablation, Atrioventricular node, Atrial fibrillation, Rate control, Pace-and-ablate, Tachycardia-bradycardia syndrome

**Key Teaching Points**

• Atrioventricular node (AVN) ablation in combination with pacemaker implantation is conventionally performed with thermal ablation. This case is the first description of the feasibility, safety, and efficacy of pulsed field ablation (PFA) for AVN ablation.
• Compared to thermal ablation, the duration of PFA is shorter, and its use has not been found to result in damage to cardiac implantable electronic devices or leads.
• Further studies are warranted to evaluate the long-term outcomes and utility of different PFA catheters for AVN ablation in patients undergoing the “pace-and-ablate” strategy.

To the best of our knowledge, this is the first reported case of the novel use of pulsed field ablation of the atrioventricular node as part of the “pace-and-ablate” strategy for rate control in atrial fibrillation.

## Case report

**Figure 1:**
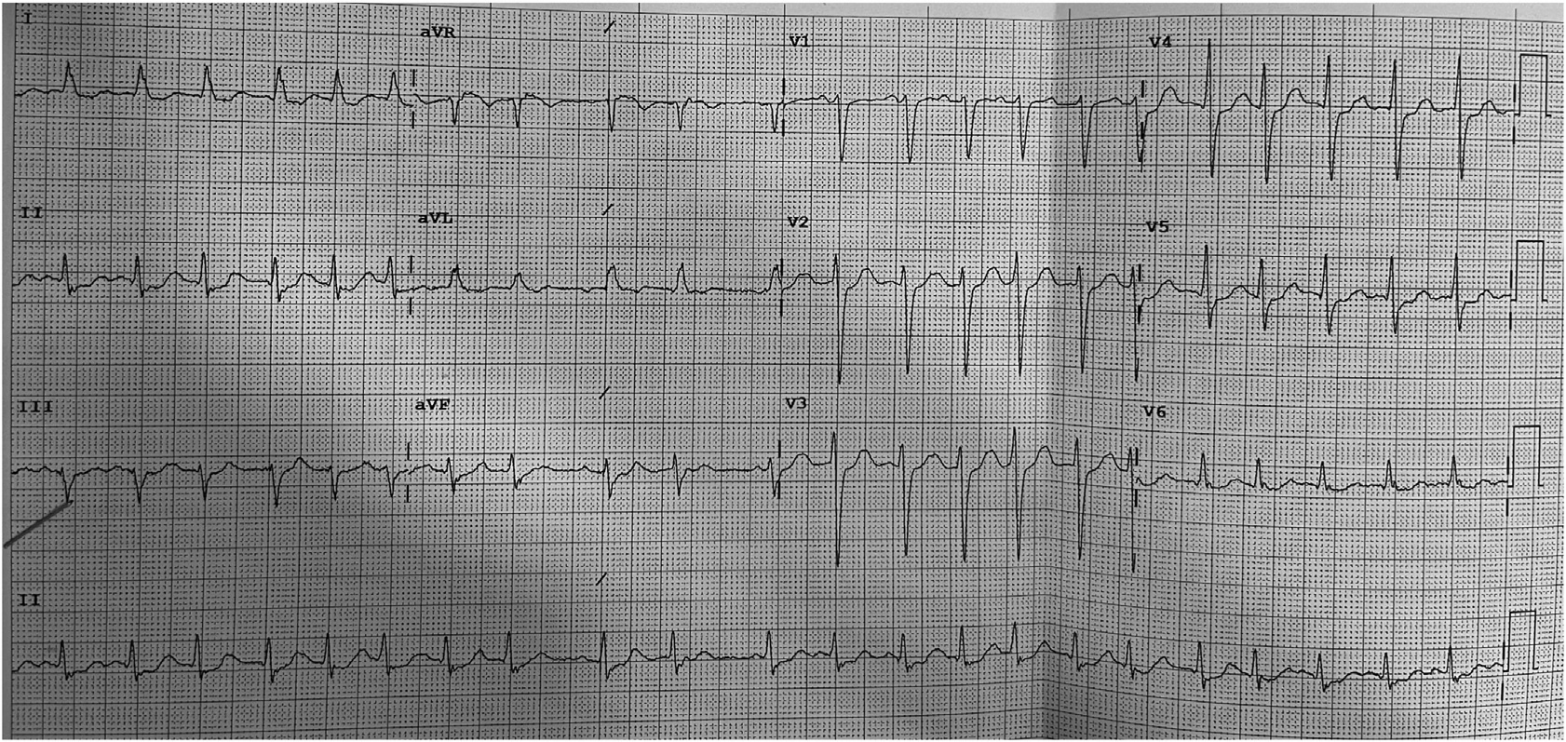
Twelve-lead electrocardiogram that documented atrial fibrillation with a fast ventricular rate of about 130 beats/min and was associated with chest pain.

An 83-year-old woman with an 11-year history of symptomatic paroxysmal atrial fibrillation (AF) and previous left atrial ablation procedures presented to our coronary care unit with chest pain secondary to paroxysmal AF with a fast ventricular response (Figure 1). This was associated with feeling unwell secondary to prolonged sinus pauses (7 seconds) upon termination of her AF episodes.

She previously underwent wide antral circumferential ablation 8 years ago (2017) for symptomatic paroxysmal AF (European Heart Rhythm Association class III symptom score with frequent hospitalizations). Her symptoms resolved and lasted for about 6 months. She was then referred to our cardiac electrophysiology clinic because of symptom recurrence, which correlated with AF and atrial tachycardia. She subsequently underwent a redo atrial ablation procedure that involved creating lateral and anterior strap lines for a documented left macroreentrant atrial tachycardia. Empirical ablation of the cavotricuspid isthmus and touch-up ablation of the left and right superior pulmonary veins and the right lower pulmonary vein were also performed in the same sitting. She remained well over the next 5–6 years until her current presentation with symptomatic paroxysmal AF.

Her other comorbidities include hypertension, hyperlipidemia, asthma, mild renal impairment, and osteoarthritis. She had a normal coronary angiogram 13 years ago (investigated for angina). She had a structurally normal heart on serial echocardiography (most recently this year). She was on uninterrupted dabigatran, bisoprolol, losartan, omeprazole, and inhalers. She previously experienced side effects from flecainide and sotalol. Her resting 12-lead electrocardiogram showed sinus bradycardia with a normal QRS duration and corrected QT interval.

During her hospital stay, she experienced frequent symptomatic episodes of paroxysmal AF and sinus pauses after termination of AF. In view of her presentation with tachycardia-bradycardia syndrome secondary to paroxysmal AF, underlying comorbidities, and limited options for medical therapy, she agreed that a “pace-and-ablate” strategy in the same inpatient setting was the most optimal treatment.

For her pacemaker procedure, the initial intention was to perform left bundle branch area pacing, but the procedure proved to be technically challenging (difficult anatomy and no satisfactory parameters could be obtained). Biventricular pacing was then attempted, but this also proved to be difficult, as pacing parameters of the left ventricular lead were unsatisfactory. In the end, she received a conventional dual-chamber pacemaker and fortuitously had a satisfactory paced QRS duration of 125 ms with right apical septal pacing.

**Figure 2:**
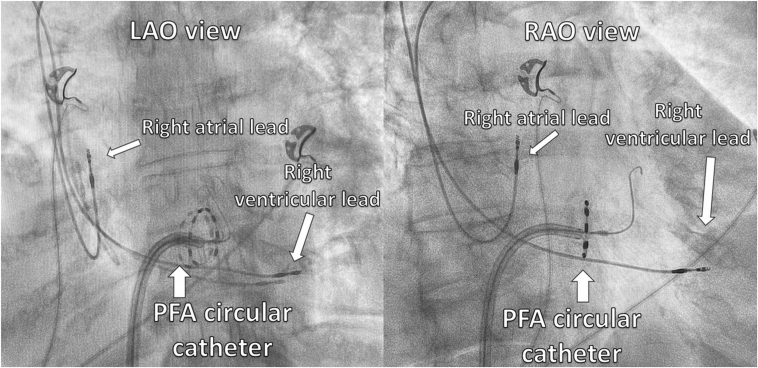
Positions of the circular pulsed field ablation (PFA) catheter (at the site of the compact atrioventricular node [AVN] location) and pacemaker leads seen in the left anterior oblique (LAO) and right anterior oblique (RAO) fluoroscopic views. The guidewire (“PV tracker”) is positioned ahead of the PFA catheter and parked in the right ventricle. Vascular access was obtained via the right femoral vein, and the circular PFA catheter was delivered through a 10-F long support sheath.

**Figure 3:**
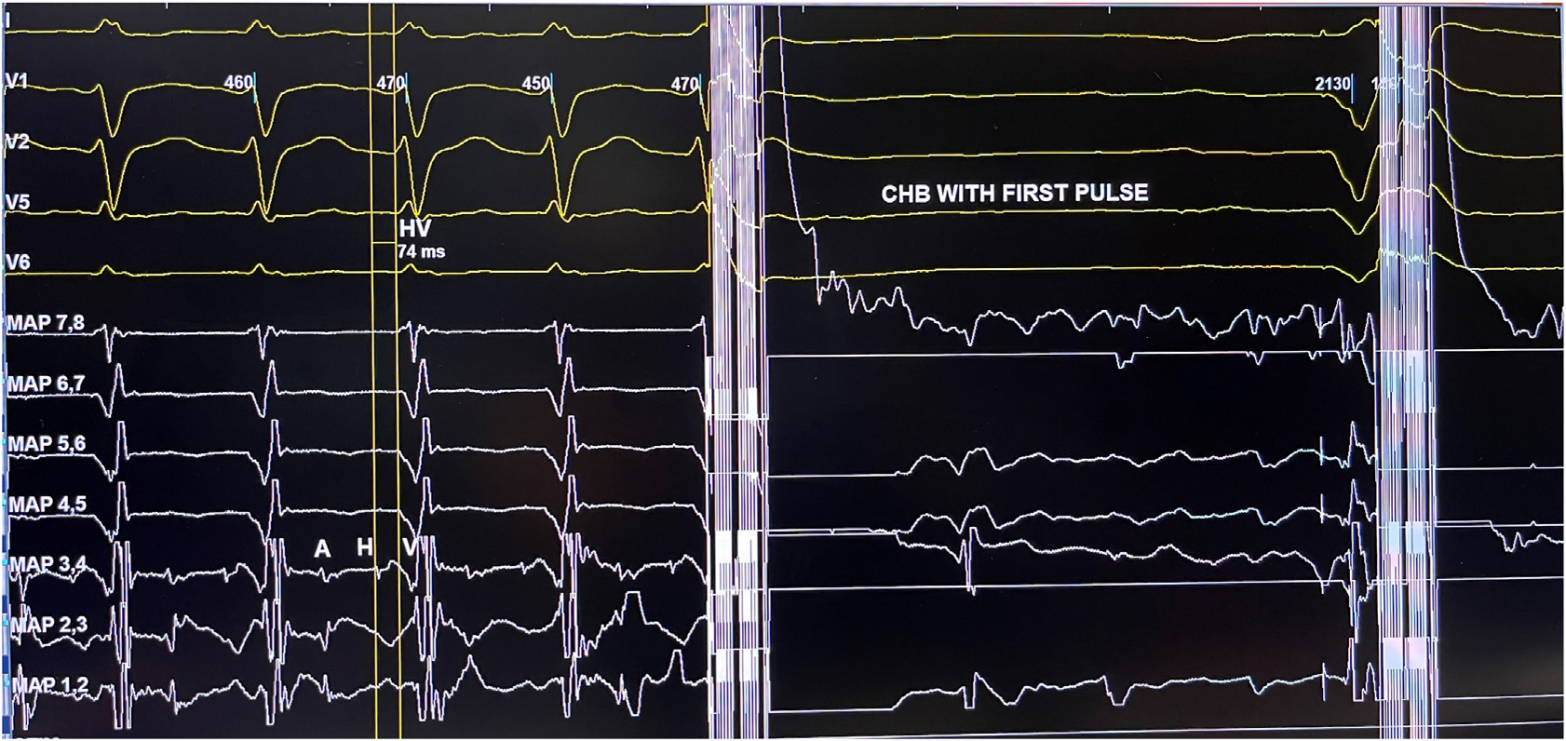
Complete heart block (CHB) (together with a paced ventricular beat) observed during the first pulsed field ablation application. The HV interval was prolonged at 74 ms. The electrogram trace is shown at a sweep speed of 100 mm/s.

**Figure 4:**
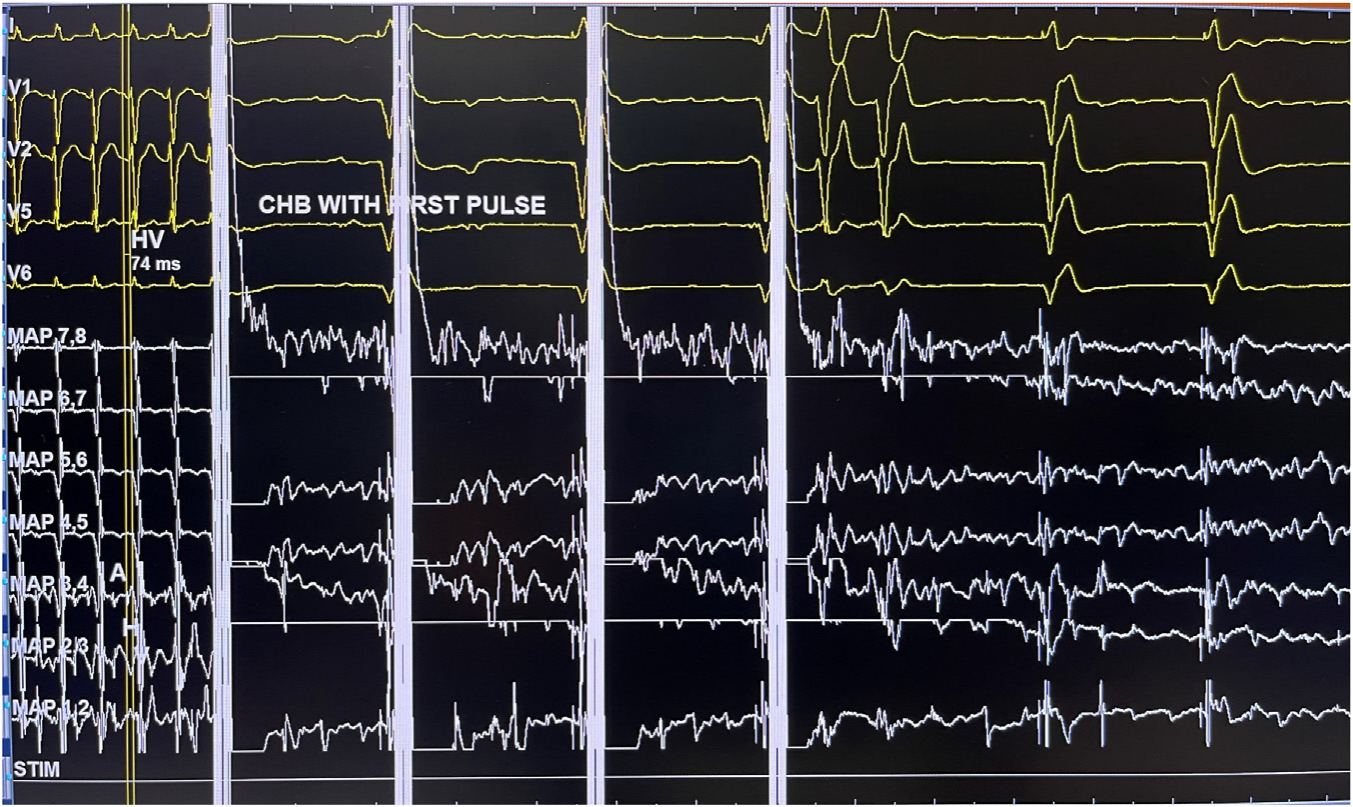
The same electrogram trace as in Figure 3 is shown at a sweep speed of 25 mm/s. Each pulsed field ablation application consists of 4 pulses (1.5 kV, each with a pulse duration of ∼150 ms). Delivery of each pulse is timed with the QRS complex (in this case, the paced QRS complex). Sustained atrioventricular dissociation is observed on the electrocardiographic leads after delivery of the first pulse. Two ventricular ectopic beats are observed after the fourth pulse, followed by ventricular pacing beats. CHB = complete heart block.

**Figure 5:**
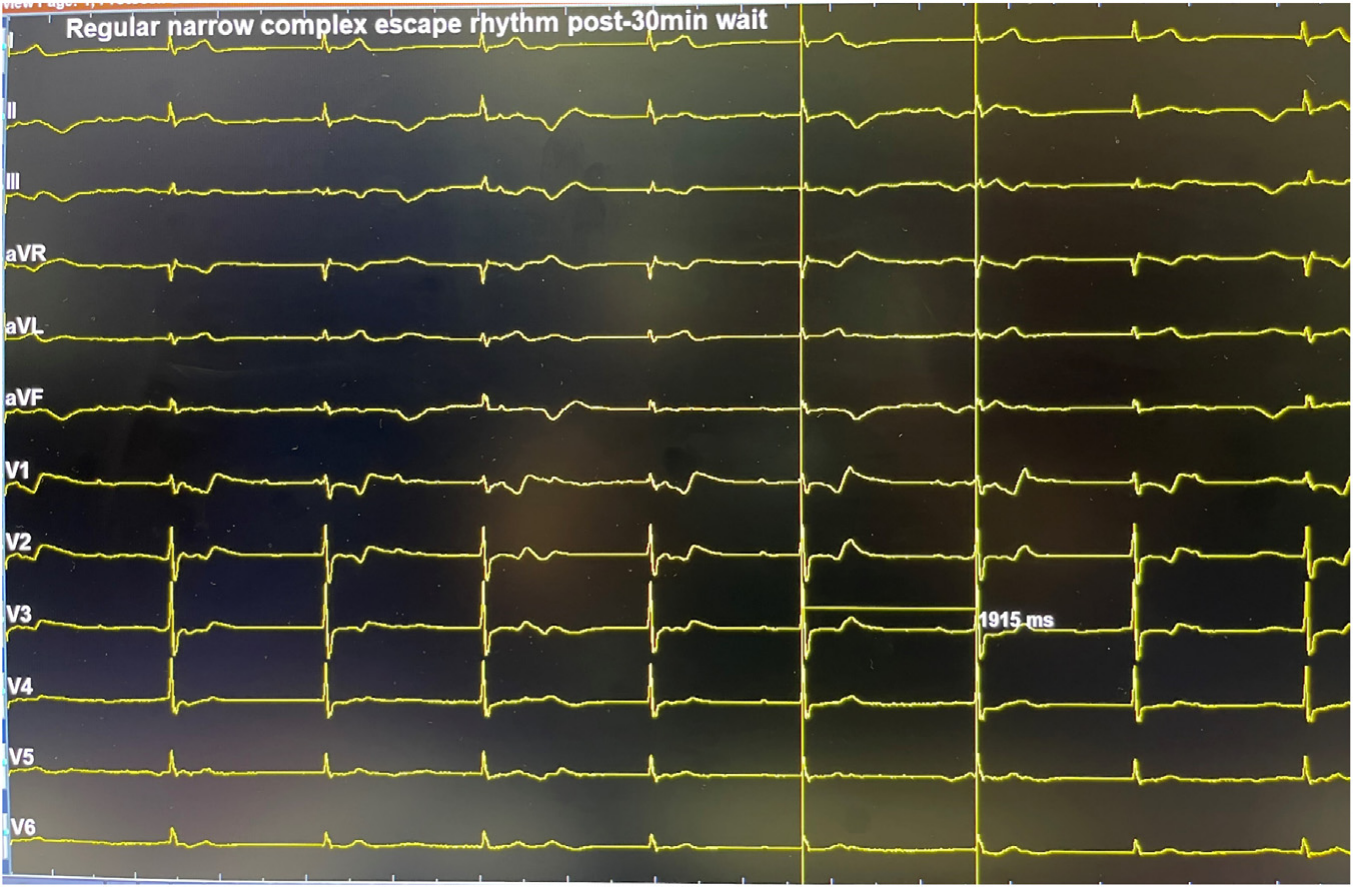
Electrocardiogram at the end of the 30-minute wait period after pulsed field ablation (PFA) demonstrating a regular narrow complex escape rhythm slightly above 30 beats/min (sweep speed 25 mm/s).

She proceeded to have her atrioventricular node (AVN) ablation the following day. As she was frail and her pacemaker procedure was challenging, pulsed field ablation (PFA) of the AVN was attempted as the procedure and ablation duration are shorter, with potentially less impact on her pacemaker leads. The procedure was performed under general anesthesia. At the start of the procedure, her pacemaker was programmed to VVI mode (base rate 30 beats/min). Under fluoroscopic guidance (Figure 2), PFA was applied to the site of the compact AVN using a 9-electrode circular catheter (PulseSelect, Medtronic, Minneapolis, MN) via a 10-F long sheath (FlexCath Contour, Medtronic). During the first PFA application, complete heart block was achieved (Figures 3 and 4). A total of 5 PFA applications were delivered, with an ablation duration of 2.9 seconds, a fluoroscopy time of 4 minutes 25 seconds, and a dose-area product of 1.49 Gy·cm^2^. There was no evidence of recovery of AVN conduction during the routine 30-minute wait period, with demonstration of a regular narrow complex escape rhythm (Figure 5). Her pacemaker interrogation postablation showed stable parameters. It was programmed to DDD mode with a lower rate set at 80 beats/min (routinely lowered to 60 beats/min at the next pacemaker follow-up visit). Coronary angiography was not performed as the target location of ablation was felt to be at a safe distance from the coronary arteries. Her 12-lead electrocardiogram was monitored for ischemic changes before, during, and after ablation (no ischemic changes were observed).

The patient made a good recovery from both procedures, attained symptomatic improvement, and was discharged 2 days after AVN ablation. Her routine pacemaker interrogation at 6-week follow-up showed 100% ventricular pacing (the underlying rhythm was AF with complete heart block), and she remained well and symptom-free.

AVN ablation in combination with pacemaker implantation is an effective strategy for patients with AF unresponsive to or ineligible for intensive rate and rhythm control therapy as per the 2024 European Society of Cardiology guidelines for the management of AF (Class IIa recommendation, Level of Evidence B).^1^ AVN ablation is conventionally performed with thermal ablation (radiofrequency ablation or cryoablation).^2^ To our knowledge, this case is the first description of the feasibility, safety, and efficacy of PFA for AVN ablation. The circular PFA catheter is primarily used for pulmonary vein isolation, and in our hands, we have recently demonstrated its versatility in treating other arrhythmias.^3^ It is worth noting that the use of a large-footprint PFA catheter for AVN ablation has the potential to deliver larger lesion sets than required, compared to a focal radiofrequency or PFA catheter, and should be used with caution. Nevertheless, compared to thermal ablation, the duration of PFA is shorter, and its use has not been found to result in damage to cardiac implantable electronic devices or leads.^4^

Further studies are warranted to evaluate the long-term outcomes of different PFA catheters for AVN ablation in patients undergoing the “pace-and-ablate” strategy.

## Disclosures

All authors declare that they have no conflicts of interest.
